# Cryopreserved mouse fetal liver stromal cells treated with mitomycin C are able to support the growth of human embryonic stem cells

**DOI:** 10.3892/etm.2014.1801

**Published:** 2014-06-23

**Authors:** WEI ZHANG, JIABO HU, QUANHUI MA, SANQIANG HU, YANYAN WANG, XIANGMEI WEN, YONGBIN MA, HONG XU, HUI QIAN, WENRONG XU

**Affiliations:** 1 School of Medical Science and Laboratory Medicine, Jiangsu University, Zhenjiang, Jiangsu 212013, P.R. China; 2 Department of Clinical Laboratory, Jiangsu Provincial Hospital on Integration of Chinese and Western Medicine, Nanjing, Jiangsu 210028, P.R. China; 3 Maternal and Child Care Service Centre of Lianyungang, Lianyungang, Jiangsu 222006, P.R. China; 4 Department of Clinical Laboratory, Zhenjiang Centre for Disease Prevention and Control, Zhenjiang, Jiangsu 212003, P.R. China

**Keywords:** human embryonic stem cells, KM3, feeder, cryopreservation, recovery

## Abstract

An immortalized mouse fetal liver stromal cell line, named KM3, has demonstrated the potential to support the growth and maintenance of human embryonic stem cells (hESCs). In this study, the characteristics of KM3 cells were examined following cryopreservation at −70°C and in liquid nitrogen for 15, 30 and 60 days following treatment with 10 μg/ml mitomycin C. In addition, whether the KM3 cells were suitable for use as feeder cells to support the growth of hESCs was evaluated. The inhibition of mitosis without cell death was observed when the KM3 cells were treated with 10 μg/ml mitomycin C for 2 h. The morphology of the KM3 cells cryopreserved in liquid nitrogen for 60 days was not markedly changed, and the cell survival rate was 84.60±1.14%. By contrast, the survival rate of the KM3 cells was 66.40±2.88% following cryopreservation at −70°C for 60 days; the cells readily detached, were maintained for a shorter time, and had a reduced expression level of basic fibroblast growth factor. hESCs cultured on KM3 cells cryopreserved in liquid nitrogen for 60 days showed the typical bird’s nest structure, with clear boundaries and a differentiation rate of 16.33±2.08%. The differentiation rate of hESCs cultured on KM3 cells cryopreserved at −70°C for 60 days was 37.67±3.51%. These results indicate that the cryopreserved KM3 cells treated with mitomycin C may be directly used in the subculture of hESCs, and the effect is relatively good with −70°C short-term or liquid nitrogen cryopreservation.

## Introduction

Human embryonic stem cells (hESCs) are derived from inner cell masses of blastocyst-stage human embryos (1), and they have an almost unlimited self-renewal ability, together with the potential to differentiate into any cell type in the body. The self-renewal capacity of hESCs is regulated by a set of transcription factors, including Oct-4, Nanog and Sox-2 (2). The differentiation of hESCs *in vitro* provides a model for studying the cellular and molecular mechanisms of early development, and hESCs may be utilized as tools for drug discovery and modeling diseases (3,4). Traditionally, the maintenance and propagation of hESCs require feeder cells, including mitotically inactivated mouse embryonic fibroblasts (MEFs) (1) or human fibroblasts (5–7), which secrete various factors that prevent hESCs from spontaneous differentiation. Several studies have focused on secreted factors released from MEF feeder layers that have the capacity to maintain the self-renewal of hESCs, and have identified a number of factors responsible for the maintenance of hESC pluripotency (8–10). Basic fibroblast growth factor (bFGF) is the key growth factor in the maintenance of undifferentiated hESC growth (11–13); therefore, hESCs are commonly cultured in medium supplemented with knockout serum-replacement (KSR) together with bFGF on inactivated MEF feeder cells. In recent years, various protocols for culturing embryonic stem cells have become available with newer trends moving toward feeder-free or serum-free culture. However, for human and mouse embryonic stem cells, fibroblast feeder layers are often used at certain phases in the culturing procedure. The feeder cells, often MEF, provide a substrate that increases plating efficiency, helps maintain pluripotency, and facilitates the survival and growth of stem cells (14).

As previously mentioned, KM3 cells display fibroblast-like morphology, have characteristics such as rapid growth and low nutritional requirements, are able to support the growth of hESCs and are a novel type of feeder cell for the long-term proliferation of hESCs in an undifferentiated and pluripotent state (15). At present, KM3 cells have been expanded for >300 passages and have continued to maintain a fibroblast-like morphology. On this basis, the purpose of the present study was to establish a type of feeder cell that is cryopreservable and that may be directly used for hESC culture, and to evaluate the effectiveness of the feeder cells as a support for hESC subculture following recovery.

## Materials and methods

### Treatment with mitomycin C

KM3 cells were respectively treated with 10, 20 and 40 μg/ml concentrations of mitomycin C (Roche Diagnostics GmbH, Mannheim, Germany) for 2 h at 37°C in 5% CO_2_ in air at 95% humidity. This treatment was initiated when the KM3 cells had reached 80–90% confluence (2 days after passage). The cells were washed with phosphate-buffered saline (PBS) five times, then treated with 0.25% trypsin/ethylenediamine-N,N,N′,N′-tetraacetic acid (EDTA; Invitrogen Life Technologies, Carlsbad, CA, USA) at 37°C for 3 min and collected by centrifugation (120 × g, 5 min). The cells were then seeded in a 6-well cell cluster multidish (Nunc, Copenhagen, Denmark) at a density of 4.0×10^5^ cells/well. The culture medium contained 90% Dulbecco’s modified Eagle’s medium (DMEM; Invitrogen Life Technologies) and 10% newborn bovine serum (NBS; Sijiqing Biotechnology Co., Hangzhou, China). The cells were cultivated for 7 days at 37°C in 5% CO_2_ in air at 95% humidity to identify the optimum concentration of mitomycin C.

### Cryopreservation and recovery of KM3 cells

Mitomycin C at a concentration of 10 μg/ml was selected for the treatment of KM3 cells that had reached 80–90% confluence (2 days after passage), by the process described above. Freezing medium, which comprised 10% dimethyl sulfoxide [DMSO; Aladdin Reagents (Shanghai) Co., Ltd., Shanghai, China] and 90% fetal bovine serum (FBS; Invitrogen Life Technologies) was added dropwise to the collected cells, which were then placed inside a Nalgene Cryo 1°C Freezing Container (Corning Incorporated, Tewksbury, MA, USA ). The freezing container was placed in a freezer at −70°C or in liquid nitrogen for 15, 30 and 60 days following gentle reduction of the temperature. At least five tubes were subjected to each cryopreservation treatment. KM3 cells that were treated with mitomycin C but which did not undergo cryopreservation served as the control. Cells cryopreserved in liquid nitrogen for 15 days following treatment with mitomycin C were designated the liquid nitrogen 15 day group; the other groups were named in an analogous manner. An exception is for the −70°C 60 day treatment; a group termed the −70°C 60 day complement group has been added, and all experiment data for cryopreservation at −70°C for 60 days were obtained from this group. The cryovials were quickly thawed in a 37°C water bath following various cryopreservation times. Fresh culture medium was added dropwise to the vials to dilute the cryoprotectants. The cells were collected by centrifugation (120 × g, 5 min) after washing in culture medium. Culture medium was added dropwise to a total of 1 ml and Trypan blue staining was then conducted. The cell suspension (90 μl) was mixed with 10 μl 0.4% Trypan blue solution, and the number of blue cells was counted within 3 min to obtain the rate of Trypan blue exclusion. The cells were seeded in a 6-well cell culture cluster at a density of 4.0×10^5^ cells/well according to the Trypan blue exclusion. The cells were cultivated for 4 days at 37°C in 5% CO_2_ in air at 95% humidity prior to the morphology of the KM3 cells being observed.

### Hematoxylin and eosin (H&E) staining and scanning electron microscopy

Cells were thawed following cryopreservation at −70°C or in liquid nitrogen for 60 days. The cells were seeded in a 24-well cell culture cluster at a density of 0.8×10^5^ cells/well according to the Trypan blue exclusion and cultivated for 4 days at 37°C in 5% CO_2_ in air and 95% humidity. The cells were fixed in 4% paraformaldehyde (PFA) for 20 min, and then H&E staining using a kit (G1120; Solarbio, Beijing, China) was conducted according to the manufacturer’s instructions. The cell morphology was observed under a scanning electron microscope (Hitachi Limited, Tokyo, Japan) (16).

### Growth curve

Cells were seeded in a 96-well cell culture cluster at a density of 0.2×10^5^ cells/well according to the Trypan blue exclusion in 200 μl medium after thawing at various times following cryopreservation by the two methods. There were five parallel wells per group. The plates were incubated at 37°C in 5% CO_2_ in air at 95% humidity for 7 days. The number of living cells was determined by MTT assay (Amresco, Solon, OH, USA), using a previously described method (17).

### Western blot analysis

Cells cryopreserved at −70°C or in liquid nitrogen for 15 or 60 days after treatment with mitomycin C were thawed, then seeded in a 6-well cell culture cluster at a density of 4.0×10^5^ cells/well according to the Trypan Blue exclusion and cultivated for 4 days at 37°C in 5% CO_2_ in air at 95% humidity. The total proteins of the cells were extracted following a previously described method (18). The total proteins were separated by SDS-PAGE and transferred to a polyvinylidene difluoride (PVDF) membrane. The membranes were blotted with primary antibodies against bFGF (BBI Antibody, Sangon Biotech, Shanghai, China) at a concentration of 1:600, and β-actin (Proteintech Group, Inc., Chicago, IL, USA) at a concentration of 1:2,000, respectively, overnight at 4°C. The membranes were then incubated with species-specific horseradish peroxidase-conjugated secondary antibodies (Santa Cruz Biotechnology, Inc., Santa Cruz, CA, USA) at a concentration of 1:3,000. Finally, the immunoblots were visualized using ECL Western blotting detection reagents (GE Healthcare, Buckinghamshire, UK).

### hESC culture

A hESC line (SHhES2) was donated by Dr Jin Ying, School of Medicine, Shanghai Jiao Tong University (19). Subculture of the hESCs was conducted used a previously described procedure (15), as follows: i) KM3 cells after thawing were seeded in a 6-well cell culture cluster at the density of 4.0×10^5^ cells/well according to the Trypan Blue exclusion. There were a large number of dead cells and relatively few adherent cells in the −70°C 60 day group. Thus, a group named −70°C 60 days complement was created in order to observe whether cells cryopreserved at −70°C for 60 days are able to support the subculture of hESCs. The term complement indicates that additional cells of the −70°C 60 day group were used to provide a confluence similar to that of the other groups according to the degree of adherence. ii) The following day, hESCs were implanted. iii) The condition of the feeder cells was observed, the cell number was counted and the differentiation of the hESCs colonies prior to passaging was analyzed. Then, hESC colonies were seeded on fresh feeder at the same rate. The same steps were repeated on passage. Finally, the expansion folds of each generation were obtained. iv) According to the growth of hESCs, they were continuously cultured for five passages on the thawed feeder cells. v) When passaged to the fifth passage, alkaline phosphatase (ALP) staining was applied to the 6-well cell culture cluster. Prior to analysis, adherent cell layers were washed twice with PBS and air dried. Staining was performed using cytochemistry staining kits (Shanghai Sun Biotech Co., Ltd., Shanghai, China) according to the manufacturer’s recommendations, with the exception of staining with hematoxylin. Differentiation of hESCs was defined as a proportion of differentiated cells in the hESC clones of >30%.

### Statistical analysis

All experimental points were performed in triplicate or quadruplicate, and all assays were repeated a minimum of three times. Normally distributed variables were expressed as means ± standard deviation (SD). For multiple group comparisons, analysis of variance (ANOVA) with Dunnett’s post test was used. All statistical analyses were performed using the SPSS statistical software package, version 17.0 (SPSS, Inc., Chicago, IL, USA). P<0. 05 was considered to indicate a statistically significant difference.

## Results

### Optimum concentration of mitomycin C

**Figure 1:**
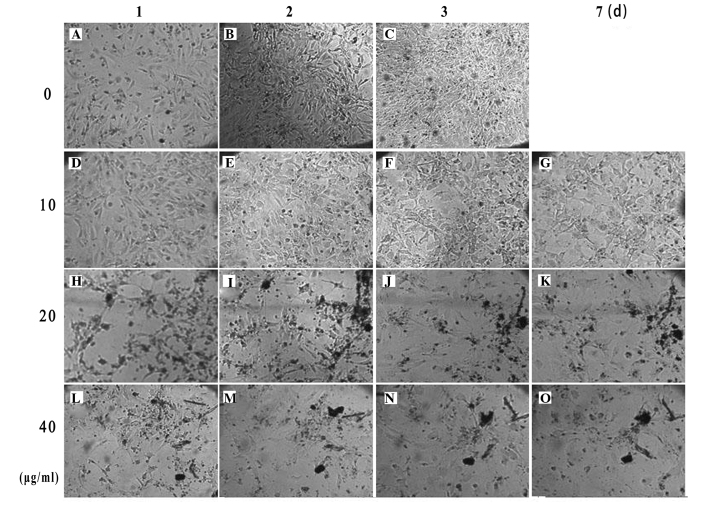
Observation of KM3 cells at different culture times following treating with various concentrations of mitomycin C for 2 h. Cell morphology after (A) 1 day, (B) 2 days and (C) 3 days for cells that were not treated with mitomycin C. Cell morphology (D) 1 day, (E) 2 days, (F) 3 days and (G) 7 days after treatment with 10 μg/ml mitomycin C for 2 h. Cell morphology (H) 1 day, (I) 2 days, (J) 3 days and (K) 7 days after treatment with 20 μg/ml mitomycin C for 2 h. Cell morphology (L) 1 day, (M) 2 days, (N) 3 days and (O) 7 days after treatment with 40 μμg/ml mitomycin C for 2 h. Magnification, ×100.

KM3 cells that were not treated with mitomycin C were highly proliferative and had reached 100% confluence on the third day after treatment (Fig. 1A–C). When the cells were treated with mitomycin C at a concentration of 10 μg/ml for 2 h, mitosis was inhibited without cell death. The feeder cells could be maintained for 7 days (Fig. 1D–G). Treatment of the cells with 20 and 40 μg/ml mitomycin C for 2 h caused a large number of cells to die after 24 h (Fig. 1H–O).

### Survival rate of KM3 cells

There were six groups in this assay and each group had five parallel wells. A summary of the cell survival rates is presented in Table I. Whether in liquid nitrogen for two months or −70°C for one month, the survival rates of KM3 cells were >80%, while the rate was only 66.40% when cryopreserved in −70°C for 60 days.

### Morphology of KM3 cells

**Figure 2:**
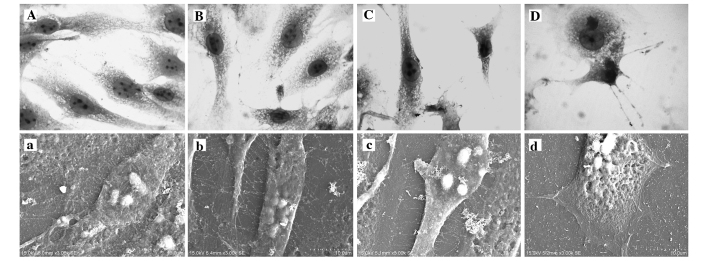
Cell morphology of mitomycin C-treated KM3 cells cultured for 4 days after thawing. Cells (A and a) not treated with mitomycin C, (B and b) treated with mitomycin C for 2 h but not frozen, (C and c) cryopreserved in liquid nitrogen for 60 days following treatment with mitomycin C for 2 h and (D and d) cryopreserved at −70°C for 60 days following treatment with mitomycin C for 2 h. (A–D) hematoxylin and eosin staining (magnification, ×1,000). (a–d) Under a scanning electron microscope (magnification, ×3,000).

The KM3 cells that had not been treated with mitomycin C were fusiform and had few cytoplasmic granules. The cell nucleus was generally oval and the karyotheca was clearly visible. It was easy to observe the nucleoli (typically 3–5). A large number of microvilli were visible under the scanning electron microscope (Fig. 2A and a). The cells in the liquid nitrogen 60 day group grew well, and were essentially the same as those in the control group with respect to morphology and growth characteristics. However, the number of microvilli was reduced and their length was shorter. After recovery, the cells adhered more slowly compared with those in the control group (Fig. 2B, b, C and c). A greater number of dead cells were observed in the −70°C 60 day group. The outwardly extending adherent cells were in a poor condition, with no typical morphology and almost no microvilli. The cells were readily detached in the rinsing process (Fig. 2D and d).

### Growth curves

**Figure 3:**
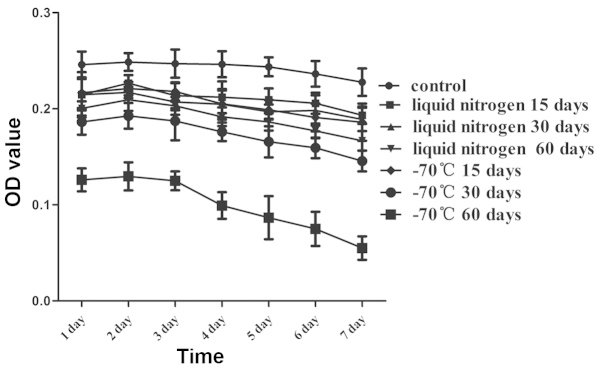
Growth curve of mitomycin C-treated KM3 cells prior to and following cryopreservation. OD, optical density.

In order to investigate the biological activity of the KM3 cells, the cells were incubated for 7 days following rapid thawing. As shown in Fig. 3, the number of adherent cells was significantly reduced in the −70°C 60 day group from the first day compared with that in the control group. In addition, the number of adherent cells in the −70°C 60 day group clearly continued to decline after 3 days. There were greater numbers of attached cells in the other thawed groups; however, these numbers were reduced compared with those in the control group. The cryopreserved KM3 cells remained stable for at least 4 days and the cell growth curves decreased slowly.

### bFGF expression levels of KM3 cells

**Figure 4:**
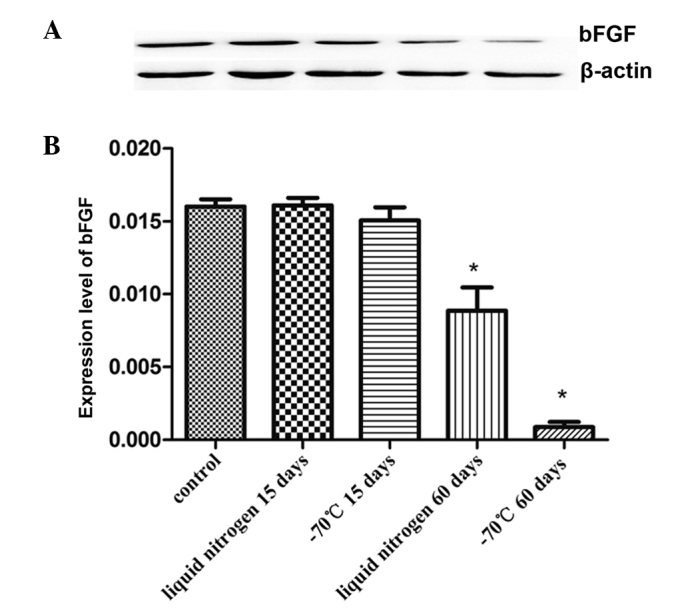
bFGF expression levels of mitomycin C-treated KM3 cells prior to and following cryopreservation. (A) Western blotting bands. (B) Analysis of the optical density of the bands. bFGF, basic fibroblast growth factor. ^*^ P<0.05 compared with the other groups..

The proteins of KM3 cells were tested to evaluate the expression levels bFGF. As shown in Fig. 4, there was no significant difference in the expression level of bFGF between the short-term cryopreservation groups (15 days) and the control group. However, the bFGF expression level fell following 60 days of cryopreservation, and the reduction in the −70°C 60 day group was particularly evident.

### Characteristics of hESCs cultured on thawed KM3 cells

**Figure 5:**
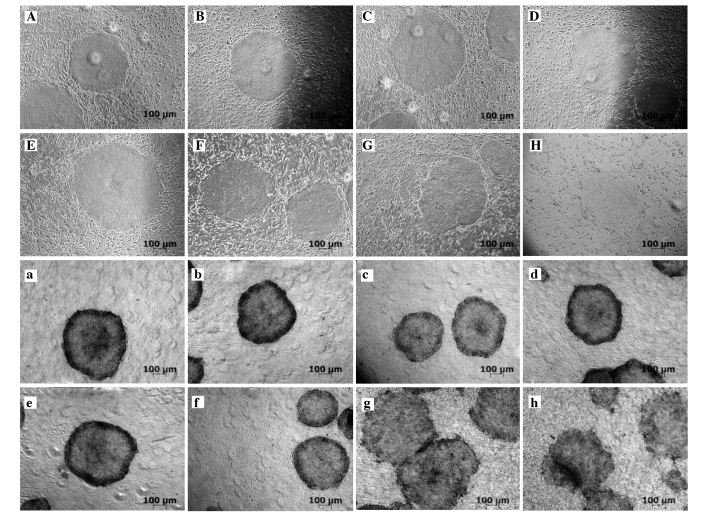
Morphology and alkaline phosphatase staining of hESCs grown on KM3 cells that had been cryopreserved and thawed after treatment with mitomycin C. (A–H) Phase microscopy. (a–h) Alkaline phosphatase staining. (A and a), control. Cryopreserved in liquid nitrogen for (B and b) 15 days, (C and c) 30 days and (D and d) 60 days respectively. Cyopreserved at −70°C for (E and e) 15 days, (F and f) 30 days, (G and g) 60 days and (H and h) 60 days with increased cell numbers, respectively. Bar, 100 μm. Magnification, ×50.

**Figure 6:**
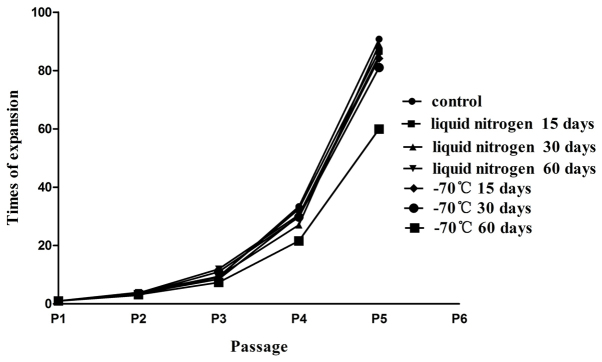
Expansion times of hESCs continuously cultured on KM3 cells treated with mitomycin C. hESCs, human embryonic stem cells.

hESCs were cultured on mitotically inactive KM3 cells. These hESCs were continuously cultured and split once every 4 days. The hESCs was then transferred from KM3 cells to recovered KM3 cells. Certain colonies continued to grow in KM3 as a control. It was observed that hESC colonies grown on liquid nitrogen 60 day feeder layers retained the typical undifferentiated morphology (round with defined colony edges) and exhibited no significant difference from the control group, as shown in Fig. 5A, a, D and d). The number of hESCs colonies seeded on the −70°C 60 days feeder layers was distinctly reduced, possibly due to the presence of dead cells resulting in a low density. Whether cell numbers were supplemented (in the complement group) or not, the majority of the hESC colonies were clearly differentiated and loosely arranged, with no clear boundary, thin clumps and morphological heterogeneity (Fig. 5G, g, H and h). A statistical analysis of the differentiation rates of hESC colonies cultivated on thawed KM3 cells was conducted and is shown in Table II. With the prolongation of frozen time, the differentiation rate of hESCs increased, particularly obvious with the rate reaching 37.67% of the −70°C 60-day group. The proliferation times of hESCs cultured on different feeders is shown in Fig. 6. The passaging ability of the −70°C 60 days complement group was less effective than that of the other cryopreservation groups.

### Feeding of hESCs

**Figure 7:**
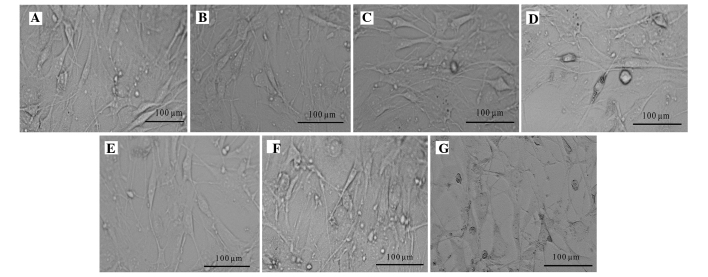
Morphology of KM3 cells treated with mitomycin C that were co-cultured with hESCs for 4 days. (A) Control. (B–D) Cryopreserved in liquid nitrogen for 15, 30 and 60 days respectively. (E–G) Cryopreserved at −70°C for 15 and 30 days, and for 60 days with complement, respectively. hESCs, human embryonic stem cells. Bar, 100 μm. Magnification, ×200.

The KM3 feeder cells supported the growth of hESCs when co-cultured with the hESCs in hESC medium for 4 days. Fusiform cells became elongated. However, in the −70°C 60 day complement group, the cell bodies were dark with a poor three-dimensional shape and there was an increased number of dead cells. The feeder cells of the liquid nitrogen 60 day group retained a better status and exhibited no clear morphological changes, as shown in Fig. 7.

## Discussion

The use of a feeder layer is one of the most commonly used methods for the culture of hESCs. Studies have shown that the proliferation of cells of the feeder layer may be reduced when they are treated with mitomycin C or exposed to radiation, but the cells remain able to survive, and are able to secrete certain cytokines required by the hESCs, such as fibroblast growth factor, insulin-like growth factor and leukemia inhibitory factor. Thus, they are able to support the subculture of hESCs (8–10).

KM3 is an immortalized cell line. Early experimental results showed that KM3 cells are able to function as feeder layers for the expansion of hESCs *in vitro*; clones of hESCs remain in the typical undifferentiated state, with maintenance of their pluripotency (15). Treatment with a mitomycin C at a concentration of 10 μg/ml for 2 h significantly inhibits the proliferation of KM3 cells, without causing cell death, and the KM3 cells are able to survive for 1–2 weeks. However, mitomycin C causes KM3 cells to die when its concentration is too high.

Cell cryopreservation is one of the main methods of cell preservation, with the −70°C freezing method and liquid nitrogen cryopreservation method being commonly used. The −70°C freezing method is simple to conduct, and can be used to freeze cells in batches; however, the activity of cells is likely to be decreased following long-term cryopreservation. Due to its lower temperature, the liquid nitrogen cryopreservation method may temporarily cause the cells to enter a non-growing state in order to preserve their cell characteristics. It also can be used to freeze cells for the long-term; however, meeting the experimental requirements is challenging due to a more complex method of operation, and the quantities of cells that may be frozen by this method are limited (20–22). hESCs are known to require precise conditions for culture and are routinely cultured in the presence of feeder cells, which provide a complex conditioning environment (23).

The preliminary results of the present study showed that a KM3 feeder layer can effectively maintain the long-term subculture of hESCs and maintain the totipotency of hESCs. On this basis, in the present study, a batch of KM3 cells treated with 10 μg/ml mitomycin C was cryopreserved by −70°C freezing and with liquid nitrogen for different time periods to observe the biological activity and ability to support a hESC subculture after rapid thawing. Different methods of freezing and various freezing times were selected for the cryopreservation of the treated KM3 cells. The recovery rate of the KM3 cells treated with mitomycin C was not statistically significantly different between the −70°C group and liquid nitrogen group within one month, and cells frozen by both methods were able to support the growth of hESCs. However, with the extension of time, the recovery rate of the −70°C 60 day group was only 66.40±2.88% after cryopreservation for two months, and the state of the cells was poor. In the −70°C 60 day group, H&E staining and scanning electron microscopy showed that the morphology of the cells was irregular, the boundaries of the nuclei were unclear and almost no clear nucleoli and microvilli structures were observed. Microvilli are associated with the ability to adhere and exchange substances (24,25); therefore, in the process of washing, the cells are readily detached due to the reduced number of microvilli. The growth state was unstable; on the first day after recovery, a large number of dead cells appeared. Although certain cells did not undergo Trypan blue staining, they were not able to adhere or adhered ineffectively. Thus, the number of adherent cells was significantly reduced when compared with the other groups when the same number of cells were seeded. Taking into account that the density of the feeder cells may influence the maintenance of hESCs (6,26–28), cell numbers were increased in the −70°C 60 day group in order to provide a number of adherent cells that was consistent with those in the other groups. Even though the cell number was supplemented, it was observed that the proliferation rate of the hESCs was slower in the −70°C 60 day group than that of the other groups from the beginning of the third generation; hESC colonies on the feeder were evidently differentiated (loosely arranged with no clear boundary, thin clumps and morphological heterogeneity). This indicates that KM3 cells treated with mitomycin C should not be stored for a long time in a freezer at −70°C. If mitomycin C-treated KM3 cells are preserved in the long-term for use as a feeder layer, the number of implanted cells should be increased. Although feeder cells preserved by this method may normally maintain the hESC subculture to some extent, the effect of long-term freezing is poor compared with that of short-term cryopreservation in a −70°C freezer and liquid nitrogen cryopreservation.

Notably, hESC colonies may partly or completely differentiate due to changes in certain factors in the process of passaging, but can be restored to the normal state by passaging following removal of the factors. This may be explained by the presence of numerous undifferentiated cells in the differentiated hESC colonies, or the differentiated colonies having retro-differentiation ability (29,30). In the current study, it was confirmed that bFGF plays an important role in maintaining the self-renewal and pluripotency of hESCs. When the −70°C 60 day group was compared with the other groups, the bFGF secretion level was markedly lower in the −70°C 60 day group, which may be a reason for the hESC differentiation that was observed. Further study of the cells from the −70°C 60 day group should be conducted to investigate whether hESC differentiation is inhibited by increasing the level of bFGF in the hESC culture medium. For the liquid nitrogen 60 day group, the state of the cells did not significantly change following cryopreservation and the cells were able to support the hESC subculture. It was observed that hESCs grown on this feeder formed typical nest-like structures with a close arrangement and clear edge boundaries. Importantly, the cells from this group were not observed to be significantly different compared with those of the control group. The feeder cells that were co-cultured with hESCs remained fusiform at the fourth day, which highlighted their biological activity.

The provision of a steady supply of qualified, homogeneous and ready-to-use feeder cells is one of the key factors for hESC research and the progression of hESC subculture studies. Cryopreserved KM3 cells that have been treated with 10 μg/ml mitomycin C may be directly used as feeder layer for hESCs after recovery, and the effect is relatively good with −70°C short-term or liquid nitrogen cryopreservation. This study provides new alternative materials and methods for the continuous stability of hESC subculture techniques.

## Figures and Tables

**Table I:** Survival rate of KM3 cells cryopreserved by different methods for various times after treatment with mitomycin C.

Time	Liquid nitrogen (%)	−70°C (%)
15 days	92.60±0.89	91.00±2.00
30 days	89.20±2.39	87.80±1.64
60 days	84.60±1.14^b^	66.40±2.88^a^,^b^

a P<0.05, compared with liquid nitrogen;

b P<0.05, compared with the 15 day group,.

**Table II:** Differentiation rate of hESCs cultured on the KM3 feeder cells cryopreserved by two different methods for various times following treatment with mitomycin C.

Time	Liquid nitrogen (%)	−70°C (%)
0 days	5.33±2.08	5.33±2.08
15 days	7.67±1.53	8.67±3.06
30 days	10.33±2.51	12.33±4.04
60 days	16.33±2.08^b^	37.67±3.51^a^,^b^

hESCs, human embryonic stem cells.

a P<0.05 compared with liquid nitrogen;

b P<0.05 compared with the 0 day group,.
